# Isolated unilateral temporalis muscle hypertrophy in a child: a case report with literature review

**DOI:** 10.1186/s12887-018-1061-7

**Published:** 2018-02-19

**Authors:** Jagath C. Ranasinghe, Chandani Wickramasinghe, Ganganath Rodrigo

**Affiliations:** 1 Department of Pediatrics, Teaching Hospital, Kandy, Sri Lanka; 2 Department of Radiology, Teaching Hospital, Kandy, Sri Lanka

**Keywords:** Isolated unilateral temporalis muscle hypertrophy, IUTMH in pediatrics, Sri Lanka

## Abstract

**Background:**

Temporalis muscle hypertrophy is a rare entity of masticatory muscle hypertrophy. All types of masticatory muscle hypertrophies have been documented of which temporalis muscle hypertrophy is one. Temporalis muscle hypertrophy is most commonly bilateral and usually associated with other types of masticatory muscles hypertrophy such as masseter or pterygoid hypertrophy. However, isolated unilateral temporalis muscle hypertrophy is extremely rare and only 9 cases have been reported to date in English literature since 1990 with only two patients less than 18 years. There is no exact etiology identified and the diagnosis is made by muscle biopsy combined with imaging study to exclude other possibilities. Age at presentation is ranges from 15 to 65 years with involvement of both sexes. We report the youngest child who is a seven year old girl with right side isolated unilateral temporalis muscle hypertrophy.

**Case presentation:**

In this patient, we discuss the youngest child with isolated unilateral temporalis muscle hypertrophy and literature review to date. The patient is a seven year old female presenting with painless swelling of the right temporalis muscle. There had no features of inflammation, trauma, neoplasm or history of parafunctions such as bruxism. The child was not complaining significantly headache or visual disturbances as well. She had undergone radiological assessment with ultrasound scan and contrast MRI. The diagnosis was confirmed by muscle biopsy which shows normal muscle architecture. She was managed conservatively with regular follow up.

**Conclusion:**

Isolated unilateral temporalis muscle hypertrophy is extremely rare in children. However this case raises the importance of considering alternative diagnoses despite the condition being rare in the pediatric population.

**Electronic supplementary material:**

The online version of this article (10.1186/s12887-018-1061-7) contains supplementary material, which is available to authorized users.

## Background

Masticatory muscle hypertrophy is a rare clinical entity involving isolated or combined hypertrophy of all groups of masticatory muscles, most commonly presenting bilaterally [1–3]. Since 1880, when the first case of masticatory muscle hypertrophy was reported, there have been several other cases reported in English literature to date. In these cases, masseter and temporalis muscles were most commonly mainly involved. Temporalis muscle hypertrophy presents most commonly as isolated bilateral hypertrophy or in association with bilateral masseter hypertrophy, however, unilateral temporalis muscle hypertrophy is rare. The exact etiology has not been identified but theoretically, parafunctional movements like bruxism have been considered [4–8]. Hypertrophy of the muscle can manifest as either painful or painless enlargement with or without headaches. This condition has been reported only in nine patients in English literature (Table 1). The age was ranging from 15 years to 65 years where included five females and four males. The youngest being a 15 year old adolescent girl, reported in 1998 [6] indicating that this condition is more confined to the adult population. In this study, we report the youngest reported case in a seven year old girl with a review of the current literature of note, this is the only pediatric patient presenting less than 10 years of age according to the best of our knowledge.

**Table 1:** Literature review of nine reported cases of IUTMH [17]

Authors & year	Patient Demographics		Race	Presentation	Site	Onset in Months	Treatment
	Years	Sex					
Wilson & Brown et al. 1990 [9]	43	F	Caucasian	Painless swelling	Rt	11	Supportive
Serrat et al. 1998 [6]	15	F	NR	Swelling, temporalis muscle contraction, limitation of mouth opening	Lt	12	Symptomatic
Isaac et al. 2000 [16]	35	M	Caucasian	Painless swelling	Lt	8	BtA
Lowry & Helling et al. 2003 [15]	45	M	African American	Swelling, recurrent headaches	Lt	12	Symptomatic
Prantl et al. 2005 [18]	48	F	NR	Painless swelling	Rt	12	Surgery
Prantl et al. 2005 [18]	57	F^a^	NR	Swelling, temporalis muscle contraction, headache	Rt	NR	BtA
Rokadiya & Malden et al. 2006 [19]	33	F	Caucasian	Painful swelling, headache	Lt	3	Amitriptyline,splint
Vordenbäumen et al. 2009 [7]	22	F	Caucasian	Painful swelling, recurrent headaches	Rt	6	Acetaminophen
Bonnie et al. 2013 [17]	17	M	Caucasian	Painful swelling, recurrent headaches	Rt	6	BtA
Katsetos et al. 2014 [13]	65	M	NR	Painful swelling	Lt	8	BtA
Present study	7	F	Caucasian	Painless swelling	Rt	2	Symptomatic

This table summarizes the all-previous cases of IUTMH reported in English literature up to date

*Rt* right, *Lt* Left, *M* Male, *F* Female, *BtA* Botulinum toxin type A administration

*NR* Not reported

^a^Same patient reported in 2005 presented 9 years later with a relapse

## Case presentation

**Fig. 1:**
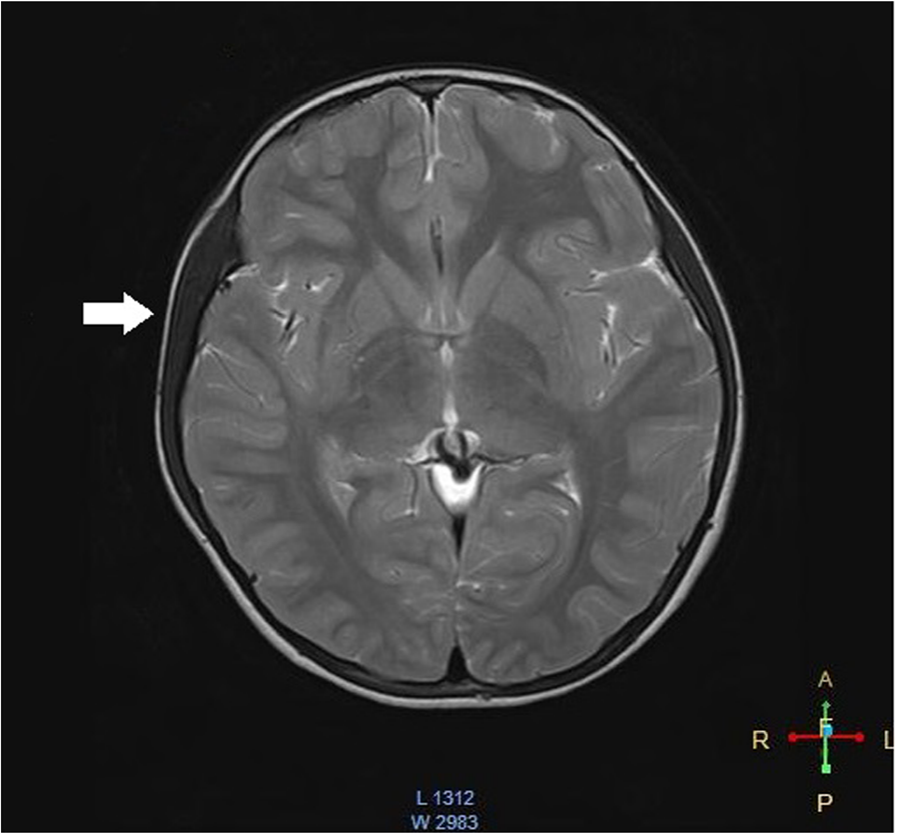
Isolated right temporalis muscle hypertrophy on an axial MRI image. T2W image of the cMRI study demonstrating enlarged right temporalis muscle on an axial section without abnormal contrast enhancement. White arrow head indicate enlarged right temporalis muscle

**Fig. 2:**
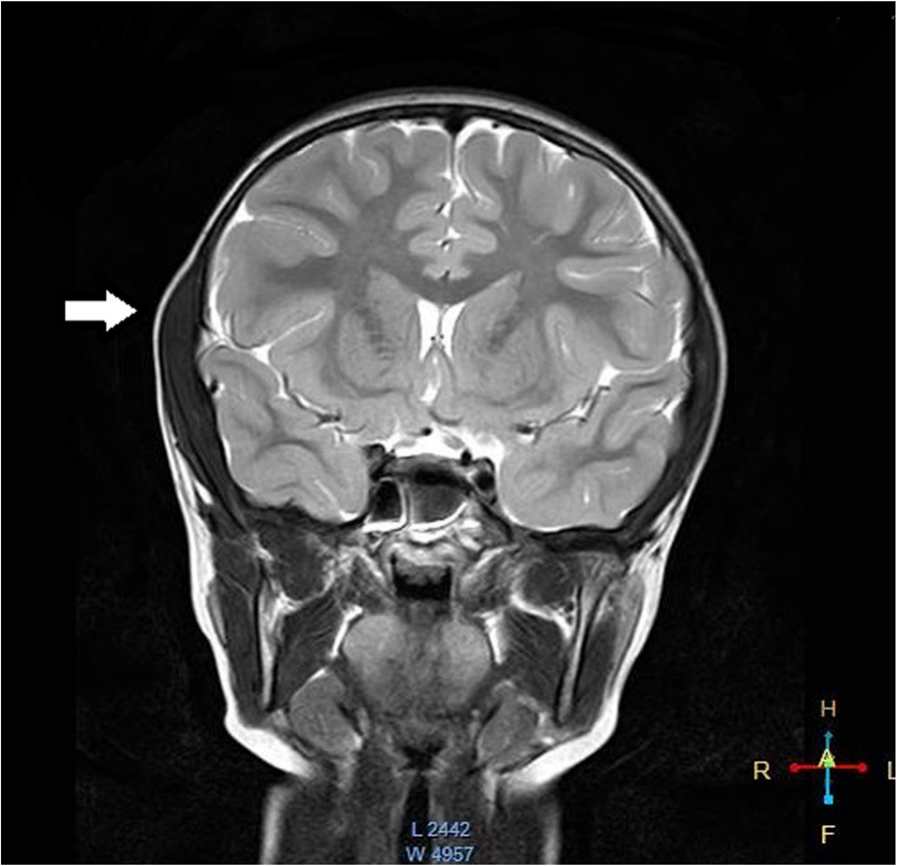
Isolated right temporalis muscle hypertrophy on a coronal MRI image. T2W image of the cMRI study demonstrating enlarged right temporalis muscle on a coronal section without abnormal contrast enhancement. White arrow head indicate enlarged right temporalis muscle

A seven year old girl presented with a lump in the right lateral forehead of two weeks duration. The mother of the patient noticed the lump while combing the child’s hair. There was no reported fever, pain, redness or tenderness over the lump indicating an inflammatory process. The patient denied any history of trauma or contact with chemicals, especially cosmetics reducing suspicion of a cutaneous hypersensitivity reaction. There was no reported visual impairment, diplopia, blurred vision, visual field defect, or opthalmoplegia to suggest ocular involvement. She strongly denied parafunction of the facial muscles like brukshism. Past medical history was non-contributory the patient reported intermittent headaches that were relieved with simple analgesics. The headache history was not compatible with migraine type or tension type headaches. The lump had not previously detected by her parents or medical practitioners. The most recent available photograph that was taken at 3 years of age revealed no evidence of hypertrophy of the right temporalis region. Physical and neurological examinations were unremarkable. The child was of average build (height 128 cm, 90th centile, weight 25 Kg, 75th centile) and healthy appearing without dysmorphism. There was marked enlargement of the right temporalis muscle, preserving the shape of the muscle, without evidence of inflammation. There was no evidence of hypertrophy of the other facial muscles on the same side or opposite side. There was no visual impairment or visual field defect. The cranial nerve examination was intact upon exam; the left side temporalis muscle was not hypertrophied. Hematological and biochemical tests were unremarkable. A complete blood count revealed a white blood cell count 9.4 × 1000 cells/mm^3^ (μL) (normal 4–12 × 1000 cells/mm^3^(μL) with 35% neutrophils and 48% lymphocytes, hemoglobin 13.2 g/dl (normal 11.5–14.5 g/dl),platelets 230 × 10^3^/mm^3^(μL) (normal 150–400 × 10^3^/mm^3^(μL), C-reactive protein 3 mg/L (normal 5 - 10 mg/L), clotting profile revealed Bleeding Time (BT) 2.5 min (normal 2–8 min), INR 1.06 (normal 0.8–1.2), activated Partial Thromboplastin Time (aPTT) 28.4 Seconds (normal 21.0–34.0 s). Liver function tests were within normal limits, including Alanine Aminotransferase (ALT) 20 U/L (normal 3–45 U/L) and Aspartate Aminotransferase (AST) 18 U/L (normal 15–50 U/L). Ultrasound Scan revealed uniformly enlarged right temporalis muscle. Cranial Magnetic Resonance Imaging (cMRI) revealed that the entire right temporalis muscle was hypertrophied compared to the left (right 9 mm vs. left 5.7 mm), preserving normal muscle morphology and normal signal intensity without abnormal contrast enhancement. No intracranial abnormalities or involvement of the bones were detected (Figs. 1 and 2). The diagnosis was confirmed by an incision biopsy, which demonstrated unremarkable skeletal muscle with preserved architecture. Available treatment modalities were discussed with the parents, and since the child was asymptomatic no immediate interventions were planned. She will be followed up regularly in the clinic (Additional file 1: Time line of events).

## Discussion and conclusion

Masticatory muscle hypertrophy is a rare clinical entity involving isolated or combined hypertrophy of all groups of masticatory muscles. Majority are bilateral with rare cases presenting as unilateral [1–3]. Isolated unilateral temporalis muscle hypertrophy is an extremely rare condition. The first case was reported by Wilson and Brown in 1990 [9]. For the last two decades, there have been only nine cases reported in English literature (Table 1). Based on the variability of age of presentation, there does not appear to be an age predilection for the disease [10]. Masseter and temporalis muscles can be involved and they may present together or in the setting of isolated, bilateral hypertrophy [2–4, 11]. The exact etiology has not been identified for masticatory muscle hypertrophy. Theoretical explanation is secondary to parafunctional jaw movements [3]. However, the other causes such as inflammation, trauma, neoplasm, myopathy, muscular dystrophy need to be excluded [2, 12]. Out of the documented cases the youngest patient with IUTMH was a 15 year old female reported in 1998 [6]. Present study is seven year old girl with IUTMH, is the youngest child reported to date according to the best of our knowledge.

Isolated unilateral temporalis muscle hypertrophy is peculiar because there is no identifiable etiology, age category or side predominance [13, 14]. The potential etiological factors for IUTMH include local factors such as bruxism, dental malocclusion, bony prominences leading to trauma and reactive hypertrophy ascribed to psychogenic factors [13, 14]. The definitive diagnosis is confirmed via histological examination of the affected muscle [14]. However, In some cases a muscle biopsy was not performed due to various reasons and patients were treated symptomatically [7]. Regarding treatment there are several treatment modalities available for IUTMH. Some patients have not undergone any active intervention and were treated symptomatically [6, 15], while others had Botulinum toxin A (BtA) injections [16, 17], surgical interventions, [18] or depending on the severity of the symptoms treatment with analgesics [7, 19]. Even though the cost associated with BtA injection is higher there are several advantages over surgical therapy. The injections are simple and less invasive, no surgical complications like trismus, fibrosis, BtA will temporally paralyze the muscle leading to atrophy, and symptomatic improvement of associated headache [13, 16–18, 20]. In this case, our patient is under regular follow up with symptomatic treatments, including analgesics and regular visual assessments. The parents were informed about the available treatment modalities and chose symptomatic treatment over other definitive treatment modalities due to the benign nature of the condition and the lack of major cosmetic concerns.

## Conclusions

IUTMH is an extremely rare condition in pediatrics and requires a high degree of suspicion as well as the exclusion of other more common etiologies of temporal swelling. This case illustrates the youngest patient with IUTMH. Initial diagnostic work up includes radiologic imaging and blood work, with the definitive diagnosis through a muscle biopsy. Based on this study and available literature, children may not need aggressive treatment, but rather require follow-up for development of further symptoms.

## Additional file

Additional file 1: Timeline of events. This data represents the time line of events carried out since diagnosis. It gives the dates and events in a chronological manner to date. (PDF 189 kb)

## Abbreviations

ALT: Alanine Aminotransferase
aPTT: Activated Partial Thromboplastin Time
AST: Aspartate Aminotransferase
BtA: Botulinum toxin A
cMRI: Cranial Magnetic Resonance Imaging
CT: Clotting Time
INR: International Normolized Ratio
IUTMH: Isolated Unilateral Temporalis Muscle Hypertrophy
