# Supplementary figures and images for: Isolated unilateral temporalis muscle hypertrophy in a child: a case report with literature review
Source: BMC Pediatr. 2018 Feb 19;18:71. doi: 10.1186/s12887-018-1061-7 (PMC5817789; doi:10.1186/s12887-018-1061-7)

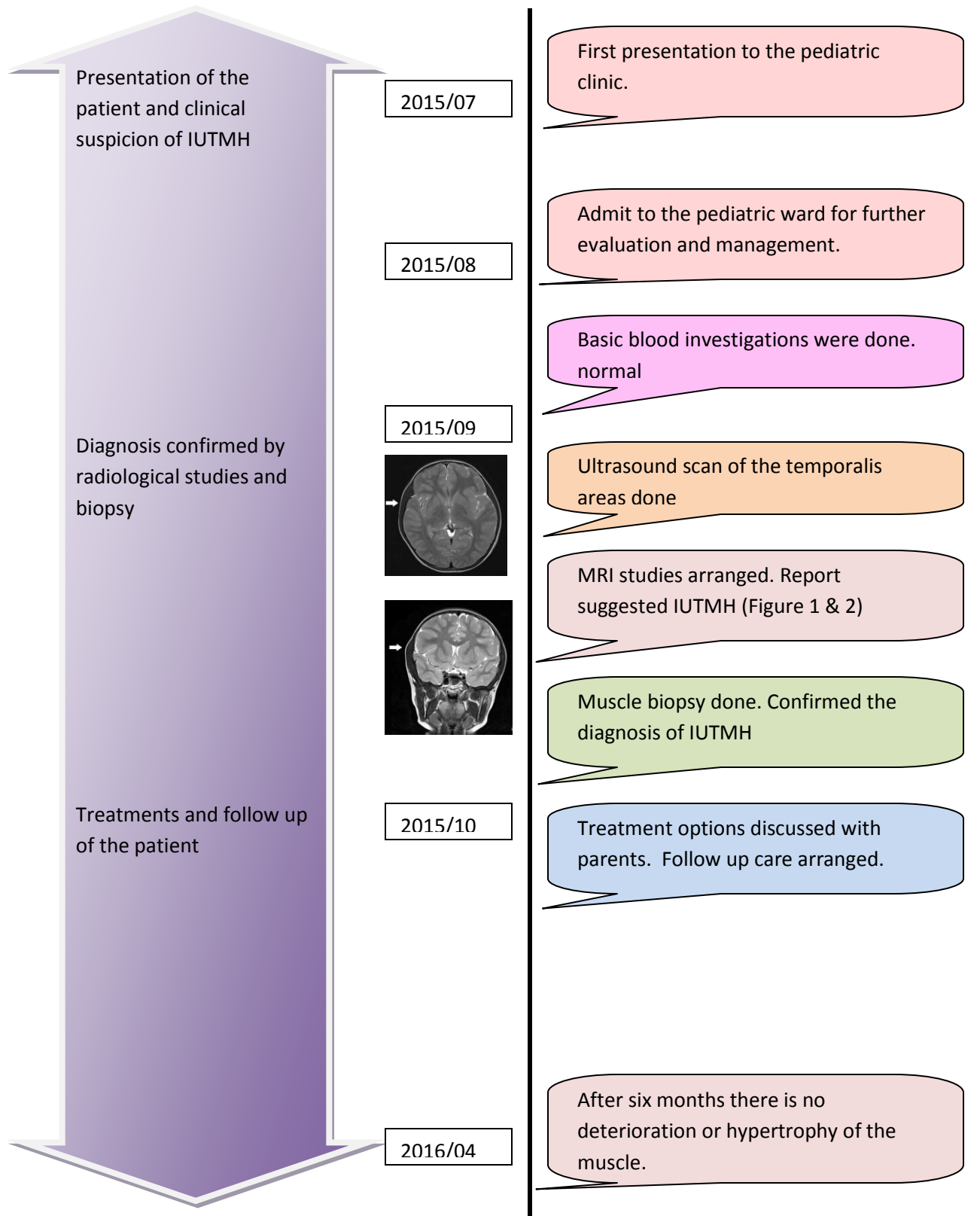

**Figure 3. Timeline of events.**

Supplement: Supplementary file 1 — Timeline of events. This data represents the time line of events carried out since diagnosis. It gives the dates and events in a chronological manner to date. (PDF 189 kb) [file 12887_2018_1061_MOESM1_ESM.pdf]
